# A Single Nucleotide Polymorphism Associated with Hepatitis C Virus Infections Located in the Distal Region of the *IL28B* Promoter Influences NF-κB-Mediated Gene Transcription

**DOI:** 10.1371/journal.pone.0075495

**Published:** 2013-10-08

**Authors:** Sreedhar Chinnaswamy, Snehajyoti Chatterjee, Ramachandran Boopathi, Shuvolina Mukherjee, Samsiddhi Bhattacharjee, Tapas K. Kundu

**Affiliations:** 1 National Institute of Biomedical Genomics, P.O.:N.S.S., Kalyani, West Bengal, India; 2 Transcription and Disease Laboratory, Molecular Biology and Genetics Unit, Jawaharlal Nehru Centre for Advanced Scientific Research, Bangalore, India; Scripps Research Institute, United States of America

## Abstract

Persistence of hepatitis C virus (HCV) infection is observed only in a subset of infected individuals and among them only some respond to treatment. Genome-wide association studies (GWAS) carried out around the world identified single nucleotide polymorphisms (SNPs) in the *IL28B* locus that are strongly associated with both HCV clearance and treatment response. The functional significance of these associations however, is not clear. In this report we show that an SNP rs28416813 in the distal promoter region of *IL28B* that is in close proximity to a non-consensus NF-κB-binding site affects downstream reporter gene expression. The effect is likely due to differential binding of NF-κB at the non-consensus site. The non-protective allele showed a reduction in luciferase reporter gene expression compared to the protective allele in HEK293T cells under different experimental conditions including treatment with tumor necrosis factor alpha (TNF-α) and 5′ triphosphorylated dsRNA. Furthermore, the HCV RNA polymerase was able to induce transcription from the *IL28B* promoter in a RIG-I-dependent manner. This induction was influenced by the alleles present at rs28416813. We also demonstrate strong linkage disequilibrium between rs28416813 and another important SNP rs12979860 in two ethnic populations. These results suggest possible mechanisms by which SNPs at the *IL28B* locus influence spontaneous clearance and treatment response in chronic HCV infections.

## Introduction

Hepatitis C virus (HCV) causes chronic liver infections affecting more than 3% of the world population [1]. Approximately 80% of patients become chronic carriers whereas the remaining 20% show spontaneous clearance. Only a fraction of the patients respond to the standard of care (SOC) treatment of pegylated interferon-alpha and ribavirin (IFN-RBV) [2]. In the year 2009 three independent groups carried out genome-wide association studies (GWAS) to identify the genes controlling the differential response to treatment against chronic HCV infections and implicated interferon-λ encoded by *IL28B* on chromosome 19 to be a key player [3]–[5]. Another GWA study carried out in the same year implicated the same gene also to be responsible for spontaneous clearance of HCV [6].

Some of the key SNPs identified in the GWAS are: rs12979860, rs8099917, rs8103142, rs28416813, rs12980275, rs8109886, rs11881222 and others [3]–[6]. The majority of the identified SNPs lie in the non-coding region of *IL28B*, whereas one SNP (rs8103142) occurs in the coding region of the protein. Among the SNPs identified by these studies rs12979860 and rs8099917 are consistently associated with treatment response in several study groups post-GWAS [7]–[11]. In fact, rs12979860 is referred to as the “cosmopolitan” SNP due to its strong associations with treatment response against HCV infections in several ethnic groups [12]. This SNP lies about 3 kb upstream of *IL28B* coding region whereas rs8099917 lies about 7.5 kb upstream of *IL28B* coding region. Some SNPs like rs11881222 have also been identified in the intronic regions of *IL28B* gene that show strong association to treatment response [5].

Despite the success of the original GWA studies there is no understanding on how any of these SNPs function to produce differential response to treatment with IFN-RBV or are responsible for spontaneous clearance of the virus. It was found that the change in the coding region of the IFN-λ3 protein caused by the variation at the SNP rs8103142 does not affect its activity in *in vitro* model systems [13], [14]. It is speculated that, since the majority of the SNPs occur in the non-coding region of *IL28B* gene including the promoter region, the SNPs may regulate its transcription [15]. However, no experimental evidence exists in favor or against this hypothesis. Therefore, we sought to examine the transcriptional role of the SNP rs28416813. This SNP was identified as one of the two potential causative SNPs in the GWAS of Ge *et al.,* [3] after sequencing the region around the most strongly associated SNP rs12979860 [15]. SNP rs28416813 was also identified by the genome-wide study of Tanaka *et al.,* [5] after sequencing the DNA flanking the region of most strongly associated SNPs that were identified on the SNP array. The ‘C’ allele at rs28416813 was in higher proportion in responders versus non-responders to SOC therapy [3], [5], [11]. Furthermore, rs28416813 has been reevaluated in other studies for its association with treatment response with IFN-RBV in chronic HCV infections [16]. In fact rs28416813 is found to be in high LD (Linkage Disequilibrium) with the “cosmopolitan” SNP rs12979860 [11]. We show evidence that this SNP influences downstream gene expression due to its proximity to an NF-κB binding element. To the best of our knowledge, this is the first evidence to functionally characterize the transcriptional role of an SNP at the *IL28B* locus relevant to chronic HCV infections.

## Materials and Methods

### Chemicals, Oligonucleotides, Patient Samples, Plasmids, Cloning and Cell Culture

All chemicals used were molecular biology grade or higher, oligonucleotides were from IDT Technologies (USA). Recombinant human tumor necrosis factor alpha (TNF-α1a) and 5′ppp-dsRNA were from Invivogen (USA). Human RIG-I and MDA5 genes in pUNO vectors were from Invivogen (USA). pUNORIG-I(K861E) and pUNO2a5BFL plasmid (encoding the RNA polymerase gene of genotype 2a HCV) were gifts from CT Ranjith-Kumar and C. Cheng Kao (Indiana University, Bloomington, IN, USA). Plasmids to express NF-κB p50 and p65 and the recombinant p50 protein expressed in *E. coli* were gifts from Mahesh Bachu, Uday Ranga lab JNCASR, Bangalore. Enzymes were either from Invitrogen or Promega. 5 ml of whole blood in EDTA was drawn from a small group of HCV (genotype 3) infected patients for genomic DNA isolation. *Ethics Statement-* Ethics clearance was obtained from the Review Committee for Protection of Research Risks to Humans of National Institute of Biomedical Genomics for the proposed work. The committee certified that the scientific aspects of the project included appropriate provision for protecting the rights and welfare of the human subjects involved. Informed consent was obtained from all participants in the study and they agreed and signed the forms to share their medical records. The above committee was satisfied with the contents and completeness of the consent forms.

The p1.4IL28B construct was amplified from patient genomic DNA (see below) using the primers IL28B1.4kbKpnFor- 5′-GATATCGGTACCCAGTGGAATTCAGGGCAAATTAC-3′OH and IL28B3.9kbHindIIIRev- 5′-GATATCAAGCTTGTGTCACAGAGAGAAAGGGAGCT-3′OH and cloned in to pGL3basic vector (Promega) at *KpnI* and *HindIII* sites. All mutations were introduced by PCR-based methods and confirmed by DNA sequencing. Human Embryonic Kidney 293T (HEK293T) and HeLa cells (ATCC, USA) were grown in Dulbecco’s Modified Eagle Medium (DMEM) supplemented with 10% fetal bovine serum (FBS). Human hepatocyte carcinoma cells (Huh7.5) were a gift from Charles Rice (Rockefeller University, NY, USA) and were cultured in media as described above along with supplemented non-essential amino acids (Invitrogen).

### Electrophorectic Mobility Shift Assay (EMSA)

HeLa cells were grown in 100 mm dishes and at 50–60% confluency were transfected with p50 and p65 expression plasmids by using Lipofectamine (Invitrogen). The cells were further grown for 6 hr and then lipopolysaccharides (LPS, Sigma) were added to the medium at a concentration of 1 µg/ml to induce translocation of NF-κB in to the nucleus. The cells were further incubated overnight and lysates were prepared 10 hr later. The cells were washed with PBS and centrifuged for nuclei and cytoplasmic extraction. A cytoplasmic & nuclear protein extraction kit was used to prepare nuclear lysates based on the protocol provided by the manufacturer (Fermentas Proteo Jet ). The following oligonucleotides were used: NF813G-TFor 5′-CCCCTGCCCTCAGTGGGCAGCCTCTGCATTCCCTCAGCTCCCTTTCTCTCTGTGA-3′; NF813G-TRev- 5′-TCACAGAGAGAAAGGGAGCTGAGGGAATGCAGAGGCTGCCCACTGAGGGCAGGGG-3′; NFC-C849For- 5′-CCCCTGCCCTCAGTGGGCAGCCTCTCCATCCCCTCAGCTCCCTTTCTCTCTGTGA-3′; NFC-C849Rev- 5′-TCACAGAGAGAAAGGGAGCTGAGGGGATGGAGAGGCTGCCCACTGAGGGCAGGGG-3′. The forward oligonucleotide (40 pm) from each pair was end-labeled with [γ^32^P]- ATP using T4 polynucleotide kinase (Promega). The labeled oligonucleotides were phenol-chloroform extracted and precipitated with ethanol. The pellets were dried and dissolved in 30 µl of 1X annealing buffer (10 mM Tris pH 8.0 and 20 mM NaCl). Equal amounts of complimentary oligonucleotides were added and annealed at 85°C and allowed for slow cooling to room temperature overnight. The probes were purified using columns packed with Sephadex G-50 and counts were obtained using a liquid scintillation counter. For each EMSA binding reaction the probe containing 5000 cpm was taken. The components of the binding reaction were: 1X Binding buffer (BB) containing 50 mM Tris (pH 8.0), 250 mM KCl, 2.5 mM EDTA,0.5% Triton X-100,62.5% glycerol(v/v), I mM dithiothreitol), the specified amount of nuclear extract,1 µl of poly dI : dc (1 µg/µl ) dissolved in 10 mM Tris/1 mM EDTA (pH 8.0) in a total volume of 10 µl. This was followed by addition of probes (1 µl of labeled probes) and the DNA binding reaction was performed at room temperature for 30 min. 2 µl of loading dye was added to each reaction mixture and run on 4% native PAGE gel for 2 hr at 4°C and dried in vacuum for 1 hr followed by exposure of the gel in Phosphorimager. EMSA with the recombinant p50 protein contained 2 µg of recombinant p50 protein instead of the nuclear lysates, but the binding reaction and other conditions were as above. Consensus NF-κB binding DNA oligos were used as competitors. Oligos H-KB(F) 5′CCGCTGGGACTTTCCAGGA-3′ and H-KB(R) 5′TCCTGGAAAGTCCCAGCGG-3′ were annealed as described above and used.

### Pull-down Assay and Western Blot

25 nm of NF813G-T For, NF813G-T Rev, NFC-C 849FOR and NFC-C849 REV primers were labeled with Biotin (IDT Technologies, USA). The complementary oligos were annealed as described for EMSA. After annealing for 5 hr at RT the oligonucleotide pairs were allowed to bind with Streptavidin Agarose (Sigma) and kept in cold room (4°C) for overnight. The tubes containing the mix were centrifuged and the supernatant was discarded. The pellet was resuspended in 1X Buffer A (20 mM HEPES, 20 mM Nacl, 5 mM MgCl2, 2 mM DTT).The mixture was centrifuged, supernatant discarded and again resuspended in 1X Buffer A. Nuclear extracts were prepared from HEK293T cells as described for the EMSA assay. The reaction used 1X BB, Streptavidin-Biotin oligonucleotides, nuclear extract, poly dI:dC and incubated at RT for 2 hours. The mixture was spun at 10,000 rpm (Eppendorf Centrifuge 5424 R) for 5 min and supernatant was collected and labeled as unbound fractions. 10 µl of loading dye was added to the agarose resin along with 10 µl of 1X Buffer A. 20 µl of 6X SDS loading dye was added to the unbound fraction and both bound and unbound fractions were boiled on hot plate at 90°C for 5 mins and run on 12% SDS-PAGE. The gels were subjected to western blot analysis and probed with antibodies against p65 (Abcam) and HRP-tagged secondary antibodies. The signal was generated by SuperSignal West Picochemiluminiscent substrate (Pierce) and exposed to autoradiography films.

### Luciferase Assays

HEK293T cells were used for reporter gene expression studies except where specified. The cells were grown in either 6-well or 96-well plates. All plasmids in the study including the *IL28B* promoter constructs which were cloned in to pGL3basic vector were run on gels to ascertain the quality and were quantified using the nanodrop spectrophotometer (Thermo Scientific, Nanodrop 8000). Cells were seeded 48 hr (6-well plates at 2×10^4^ cells/well) or 24 hr (Corning, clear bottom white 96-well plates at 2×10^4^ cells/well) prior to transfections. Lipofectamine LTX and Plus reagent (Invitrogen) was used for transfections. 500 ng of the firefly luciferase construct along with 1.5 µg of p50+p65 plasmids were used per well for the 6-well plate assays. The firefly luciferase activity was measured 24 hr after transfection in a liquid scintillation counter for 6-well plate assays. For the 96-well assays *Renilla* luciferase encoded plasmid pRLTK was used as a transfection control at 0.01 ng/well. When using Huh7.5 cells pRLCMV (where the *Renilla Luciferase* gene is driven by the cytomegalovirus, CMV promoter) was used as transfection control at 0.1 ng/well. 10 ng of the firefly luciferase (unless otherwise specified) construct along with 25 ng each of the p50 and p65 plasmids per well were used for the 96-well plate assays. Luciferase activity was measured 24 hr after transfections. Recombinant TNF-α at indicated concentrations was added in to the media 24 hr post-transfection and luciferase activity measured after 8 hr. dsRNA was transfected using lipofectamine 2000 (Invitrogen) 24 hr after transfecting the DNA plasmid constructs following the manufacturer’s instructions and luciferase assays were carried out after a further 12 hr incubation of the 96-well plates at 37°C and 5% CO_2_. 96-well plate assays used the Promega Dual Luciferase assay kit and quantified in a Glowmax 96-well plate reader (Promega) with dual injection system. All the firefly luciferase values were normalized to *Renilla* luciferase values.

### Genotyping of SNPs from Blood Genomic DNA and Generating LD Plots

Genomic DNA was isolated from whole blood using the Qiagen medi prep kit. The SNP rs12979860 was amplified using the following set of primers: Forward Primer: 860For242bp (5′-AGGCTCAGGGTCAATCACAG-3′) and reverse primer: 860Rev242bp (5′GCTTATCGCATACGGCTAGG-3′) to obtain a 240 bp amplicon. The SNP rs28416813 was amplified with the Long-range PCR kit from Qiagen by using: Forward primer 1.9kbrs813KpnIFor2 (5′GATATCGGTACCTGCATTGTACGACCCTCCAAC-3′) and reverse primer: 1.9kbil28b12aaHIIIREV1 (5′GATATCAAGCTTCAGCACTGCGGCCATCAG-3′). Due to high sequence homology between *IL28A* and *IL28B* the primers gave rise to two amplicons of sizes 2673 bp corresponding to promoter of *IL28A* and 1975 bp corresponding to that of *IL28B*. The products were run on 1% EtBr-stained agarose gels and the *IL28B* amplicon was excised from the gel and purified using Qiagen gel extraction kit. The amplicons were sequenced with internal primers using the Big dye terminator (Life tech.). The SNP rs8099917 was genotyped by RFLP. PCR was carried out using the primers: a) 917FOR700bp (5′CAAGTCAGGCAACCACATGC3′) b) 917REV 700bp (5′ GCTCTGTCTGTCTCAATCAATC-3′).The amplicon (700 bp) was pursued for RFLP. The DNA was incubated overnight(12–18 hours) with restriction enzyme *BsrDI* (New England Biolabs).The samples were run on 2% agarose gel and checked with a 100 base-pair ladder.

Genotype data of 80 CEU (Utah residents of northern and western European ancestry) individuals was downloaded from the 1000 genomes project [17] database and loaded into R using the Bioconductor [18] package VariantAnnotation. LD values (D′, r2) and plots for the 1000 genomes data as well as our data, were generated using R [19] packages genetics and LDheatmap [20] respectively.

## Results

### SNPs at the Distal Promoter Region of *IL28B* Affect Binding of NF-κB

**Figure 1:**
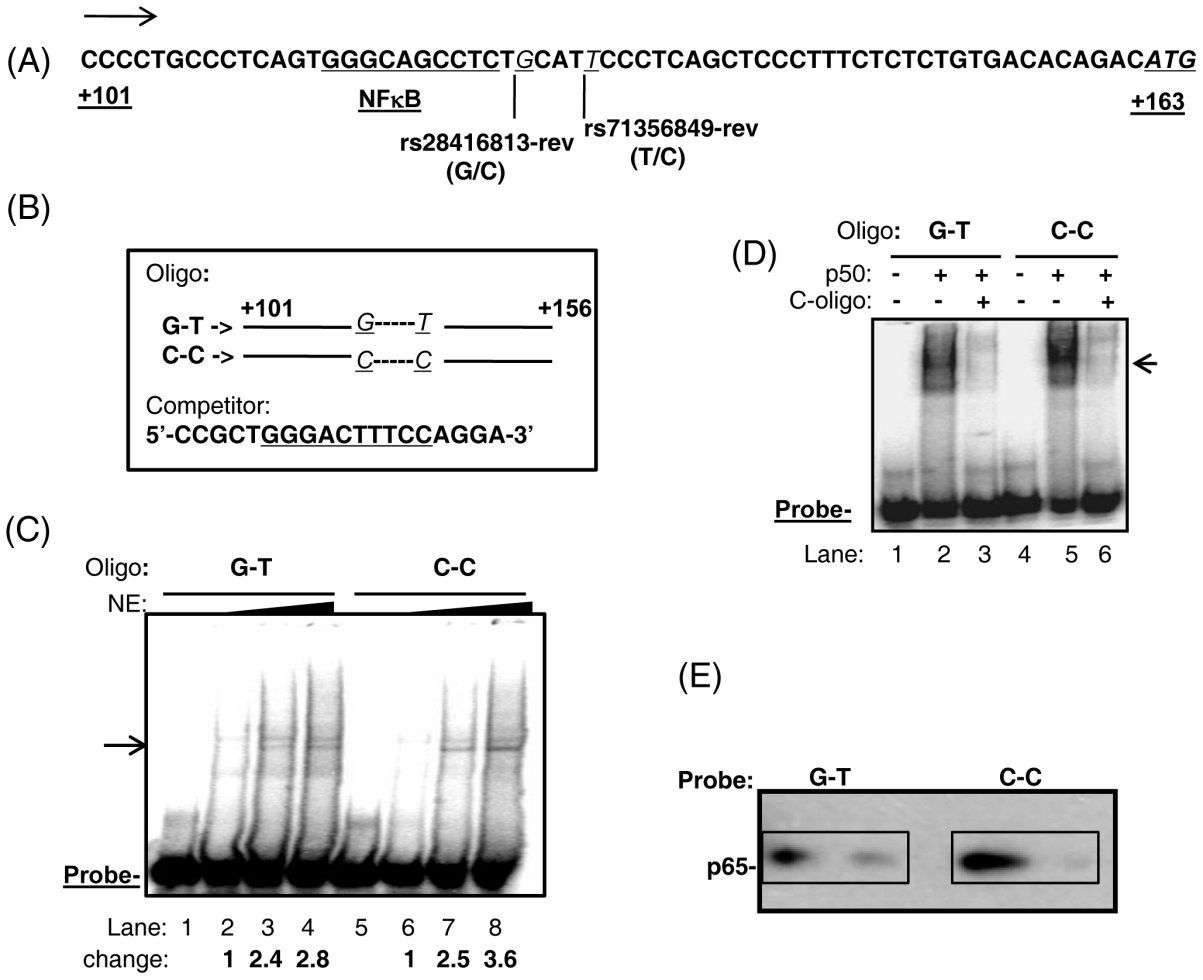
The alleles at rs28416813-rev and rs71356849-rev affect NFκB binding in EMSA. A) The distal region nucleotide sequence of the *IL28B* promoter is shown along with the two SNP positions (italics, underlined) and the non-consensus NFκB-binding site (underlined) identified by Osterlund et al. (2007) [21]. The start codon is underlined and is in italics. The numbering is with respect to TSS. Note that the naming is on the opposite DNA strand to that used for naming SNPs in dbSNP, hence the SNPs have a “rev” notation. B) The oligonucleotides used for EMSA and pull-down experiment. The consensus NF-κB binding sequence is underlined in the competitor oligonucleotide. C) Autoradiograph of EMSA carried out from nuclear extracts prepared from HeLa cells after stimulation for NF-κB signaling and overexpression. Increasing concentrations of the extracts (0, 2, 4 and 6 µl respectively in lanes 1–4 and 5–8) were used at a constant concentration of the probe. The arrow indicates a likely NF-κB dimer. The fold-change in binding at the band positions (arrow) for the two probes G-T and C-C are shown below each lane. D) EMSA with recombinant p50. Arrow indicates the shifted probe position due to binding with p50. C-oligo- competitor oligo. E) Pull-down assay and western blot. The streptavidin-bound biotin labeled probes were incubated with nuclear extracts from HEK293T cells overexpressed with activated NF-κB. The bound proteins were probed with antibodies against p65 (Abcam).

The SNP rs28416813 has been reported by GWAS to influence treatment response to HCV infections [3], [5]. This SNP exists as either a guanylate (G) or a cytidylate (C), whose frequency depends on the population being studied. Since the naming of the SNPs in the dbSNP (NCBI) is in the reverse orientation to the directionality of *IL28B* gene, we will name the SNPs with a “rev” (for reverse) notation in this report whenever we are referring to the SNPs in the same orientation as *IL28B* gene (Fig. 1A). The SNP rs28416813-rev is located downstream of the transcription start site of *IL28B* gene (TSS, as per Genebank, NCBI) at position +126 (Fig. 1A). rs71356849-rev exists three nucleotides downstream of rs28416813-rev and is either a adenylate (A) or a guanylate (G) (dbSNP, NCBI) (Note that the naming in Fig. 1A is on the opposite strand to that named in dbSNP). A closer examination of this region reveals a non-consensus NF-κB-binding site reported by Osterlund *et al.,* (2007) [21] only one base upstream of rs28416813-rev (Fig. 1A). NF-κB is a transcription factor activated by post-translational modification by several immunological pathways and functions as a homo or heterodimer of five different proteins (p50, p52, p65, Rel-B and C-Rel) [22], [23]. Activated NF-κB translocates from the cytoplasm in to the nucleus [22]. Osterlund *et al*., (2007) [21] identified the putative NF-κB-binding site by bioinformatic analysis and further confirmed its binding to NF-κB. We were interested to investigate if the alternate alleles (G/C) present at the SNP rs28416813 would affect NF-κB binding. Given the proximity of rs71356849 to rs28416813, the former was included in the examination for effects on NF-κB binding. Electrophoretic mobility shift assay (EMSA) to examine NF-κB binding was performed with a 55-mer oligonucleotide that included the non-consensus NF-κB-binding site and the alternate alleles at the two SNPs under study: G-T for G allele at rs28416813-rev and T allele for rs71356849-rev; C-C for C allele at both the SNPs in Fig. 1B. Nuclear extracts (NE) prepared from HeLa cells containing overexpressed and activated NF-κB were used in the assay at increasing concentrations. Fig. 1C shows that both the radiolabeled probes G-T and C-C bound to nuclear proteins and at least two distinct bands were visible at the three concentrations of the NE used. However, minor differences were evident in binding between the two probes. The probe C-C showed slightly increased binding at one band position when compared to probe G-T that increased with increasing concentration of the NE (Fig. 1C, arrow).

**Figure 2:**
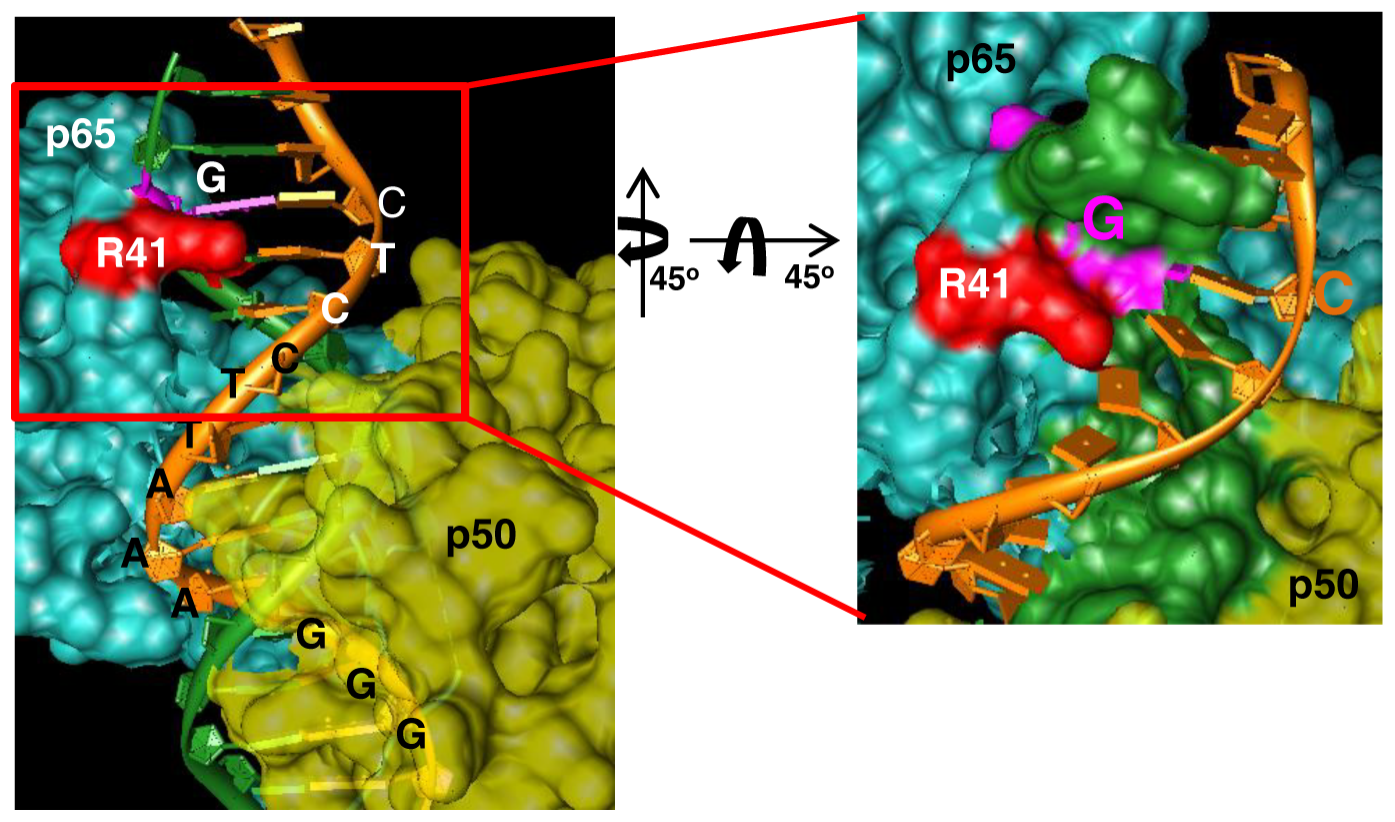
The 12^th^ base contacts R41 in p65. The 12^th^ base in the DNA equivalent to the position of rs28416813 in the IL28B promoter contacts R41 of p65 in the co-crystal structures (PDB ID 2O61). The figure generated by UCSF CHIMERA (available on the World Wide Web) shows that arginine 41 (red) of the p65 protein (in cyan, whereas p50 is in yellow) is in close contact (<3 angstroms) distance from the nucleotide at position equivalent (12^th^ position) to the SNP rs28416813 (magenta). The DNA strands are colored green and orange. A consensus oligonucleotide is used in the co-crystal structures.

EMSA with recombinant p50 protein was performed to confirm the results with nuclear extracts (Fig. 1D). Similar to the results obtained with nuclear extracts, both the probes interacted with p50, but the C-C probe moved as distinct bands as opposed to a diffuse binding pattern by the G-T probe (Fig. 1D, compare lanes 2 and 5). Furthermore, binding of p50 to both the probes was reduced by a competitor oligonucleotide that contained a consensus NF-κB-binding site (Fig. 1D, lanes 3 and 6). Binding of p65 to the probes was confirmed by a biotin-streptavidin based pull-down assay followed by western blot (Fig. 1E). These results establish the NF-κB-binding site at position +115 to +124 in the *IL28B* distal promoter region and further reveal that NF-κB binding at this site can be affected by the nucleotides present in the nearby SNPs rs28416813-rev and rs71356849-rev. We speculate that the overall low level of protein binding in both the EMSA experiments is due to the non-consensus nature of the NF-κB element. However, the above results show that the flanking sequences can influence the binding affinity of NF-κB to the non-consensus site. Molecular modeling based on the co-crystal structure of the NF-κB protein complexed to its cognate site revealed that the nucleotide that represents the position occupied by the SNP rs28416813 could contact amino acid R41 in p65 (Fig. 2).

### *IL28B* Promoter is Responsive to NF-κB

**Figure 3:**
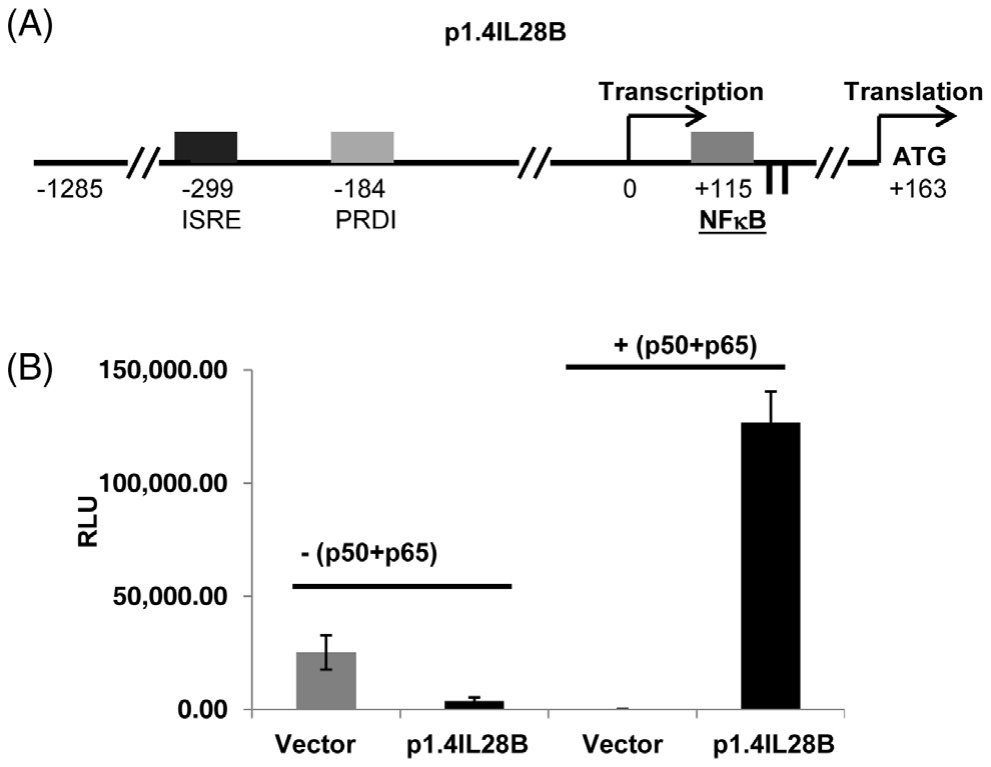
The distal fragment of the *IL28B* promoter is responsive to NF-κB. A) A schematic of the 1.4 kb fragment of the *IL28B* promoter fragment cloned in to pGL3basic vector. The rectangles show the different transcription factor binding sites identified by Osterlund et al. (2007) [21]. The vertical lines next to the non-consensus NF-κB site indicate the position of two SNPs rs28416813 and rs71356849 respectively. ISRE, Interferon-stimulated response element, PRDI, positive-regulatory domain I. The TSS is shown by the arrow before the non-consensus NF-κB-binding site (at position +115 with respect to TSS). B) Luciferase assays carried out with HEK293T cells in 6-well plates. The transfection of the p1.4IL28BC-T construct was done either in presence or absence of p50+p65 and activity compared with the empty pGL3basic vector. The error bars show SD from triplicates.

A reporter gene construct was designed to include the 1.4 kbp promoter region of *IL28B* (p1.4IL28B) (Fig. 3A). The DNA was cloned from a HCV patient who was homozygous at both rs28416813-rev and rs71356849-rev SNPs, respectively, for the C and T alleles. The DNA also included the distal promoter NF-κB-binding site (at +115) and the other transcription factor binding sites identified by previous studies [21], [24]. This construct, to be named p1.4IL28BC-T was transfected in to HEK293T cells and was assayed for luciferase expression. No luciferase expression was detected in cells transfected with p1.4IL28B when compared to vector control (Fig. 3B). Overexpression of p50 and p65 expression plasmids, however induced a high level of luciferase activity from p1.4IL28BC-T when compared to vector control, suggesting that p1.4IL28B was responsive to NF-κB within the cells. This construct was used to examine whether alternate alleles in the *IL28B* promoter can affect transcription under different experimental conditions.

### The Non-protective C Allele at rs28416813-rev Reduces Transcription of Reporter Gene in Presence of NF-κB

**Figure 4:**
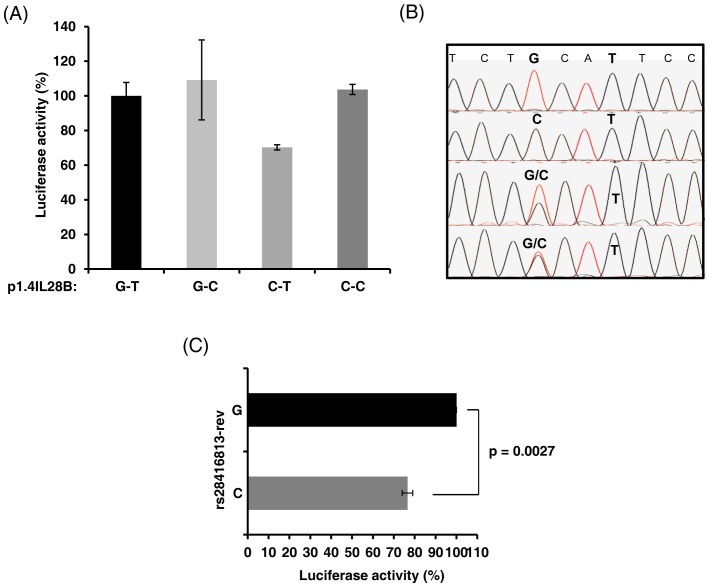
The C allele at rs28416813-rev in *IL28B* promoter decreases luciferase expression. A) Luciferase activity with p1.4IL28B constructs shows that the C allele at rs28416813-rev when present along with the T allele at rs71356849-rev decreases reporter gene expression. The ratio of firefly to *Renilla* luciferase values for the other three constructs was normalized to that of p1.4IL28BG-T construct as % activity and plotted. The data is representative of at least two independent experiments with the error bars showing SD of triplicates. B) A representative chromatogram from a small cohort of HCV infected patients (n = 25) who were sequenced at the two SNP positions (shown in bold) and only rs28416813-rev was dimorphic (C/G) with a minor allele frequency (MAF) of 0.3 whereas none of the samples showed any variation at rs71356849-rev (T). C) The C allele at rs28416813-rev consistently shows a reduction in luciferase activity by 20–30%. Results show the mean (76.5) of four independent experiments where samples were processed in triplicate and were controlled for transfection efficiency and were normalized to the G allele. Error bars show SE.

Using p1.4IL28BC-T we introduced mutations comprising the alternate alleles at both SNP positions respectively to generate constructs: p1.4IL28BG-C, p1.4IL28B G-T and p1.4IL28B C-C. The four plasmid clones were transfected in to HEK293T cells along with both p50 and p65 encoded expression plasmids, and assayed for reporter gene expression. P1.4IL28BC-T showed a reduction in luciferase activity compared to p1.4IL28BG-T. Constructs p1.4IL28BG-C and p1.4IL28BC-C had comparable activity as p1.4IL28BG-T (Fig. 4A). Therefore, the C allele at rs28416813-rev decreased downstream reporter gene expression when accompanied by a T allele at rs71356849-rev.

**Figure 5:**
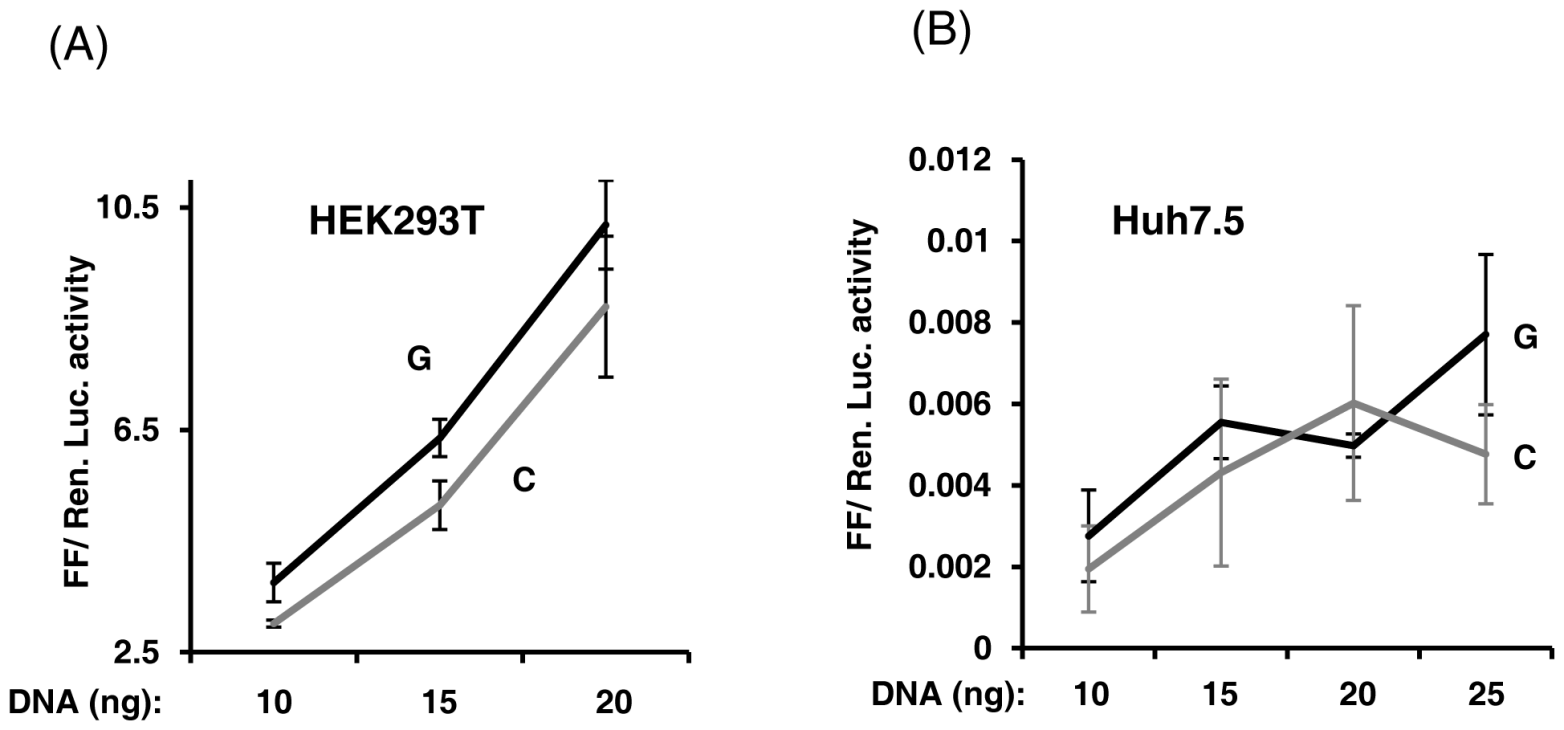
The p1.4IL28B construct is not responsive to exogenously provided NF-κB in Huh7.5 cells. A) p1.4IL28B constructs carrying either G or C alleles at rs28416813-rev were transfected in to HEK293T cells at increasing concentrations while plasmids encoding p50 and p65 were held constant at 50 ng/well. The C allele at rs28416813-rev decreases transcription at different p1.4IL28B plasmid concentrations. B) Under similar conditions the p1.4IL28B construct does not show robust reporter gene activity in Huh7.5 cells. The error bars show SD of triplicates. This experiment was confirmed at least two times at different pRLCMV concentrations.

We genotyped a cohort of HCV infected patients and found that only rs28416813-rev was dimorphic (G/C) with a minor allele frequency (MAF) of 0.3 whereas rs71356849-rev was monomorphic (T) in our studied population (Fig. 4B). We therefore sought to further examine the effect of only rs28416813-rev on downstream gene expression. We observed a consistent 20 to 30% reduction in luciferase activity involving the C allele at rs28416813-rev in comparison to the G allele in cells transfected to express 25 ng of NF-κB and 10–15 ng of p1.4IL28B constructs (p = 0.0027, two-tailed Student’s t-test) (Fig. 4C). The difference in activity was evident at different p1.4IL28B plasmid concentrations (Fig. 5A). We next used the Huh7.5 cells that can support efficient HCV replication [25], [26] in culture to test for the responsiveness of the p1.4IL28B construct to exogenously provided NF-κB (Fig. 5B). However, unlike the HEK293T cells, the Huh7.5 cells did not show a robust stimulation of the *IL28B* promoter activity even at higher DNA concentrations (25 ng plasmid DNA) (Fig. 5), although the CMV promoter driven *Renilla Luciferase* expression was robust. We currently do not know the reason for this poor transcriptional activity of the p1.4IL28B promoter in Huh7.5 cells. Huh7.5 cells are known to be deficient in the retinoic acid inducible gene-I (RIG-I) pathway [26] and it can be speculated that this pathway is critical for *IL28B* promoter activity. This also shows that the IFN-λ3 enhanceosome components include factors other than p50 and p65, possibly IRF-3 (interferon regulatory factor 3) which is not appropriately activated in Huh7.5 cells [26].

### The Non-protective C Allele at rs28416813-rev Reduces Transcription of Reporter Gene likely by Decreasing NF-κB Binding at the Non-consensus Site

**Figure 6:**
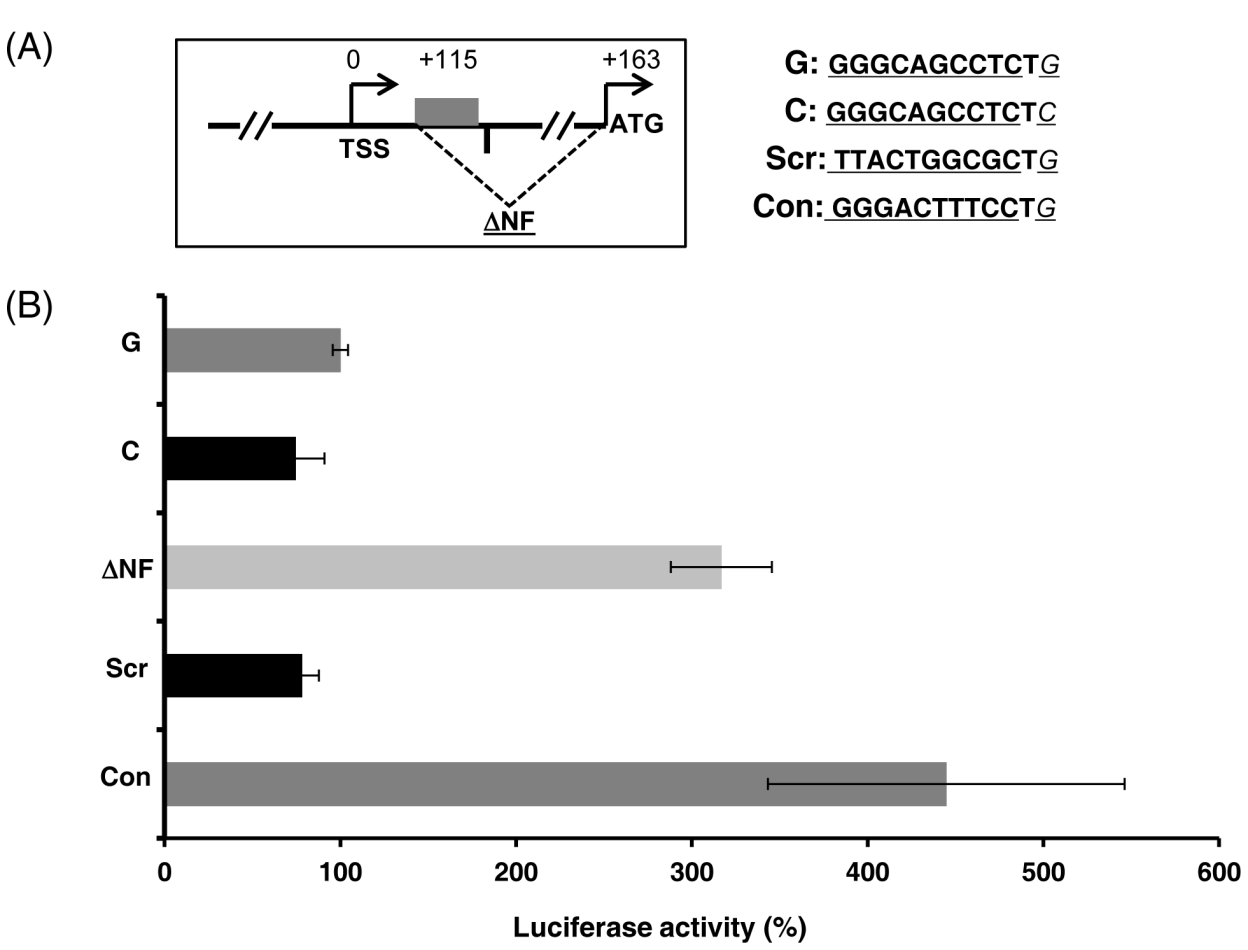
The non-protective C allele at rs28416813-rev reduces transcription of reporter gene likely by decreasing NF-κB binding at the non-consensus site. A) A schematic of the ΔNF construct made with the p1.4IL28B construct with G allele at rs28416813-rev. TSS-transcription start site. The grey rectangle represents the non-consensus NF-κB-binding site between +115 to +124. The vertical line next to the grey rectangle denotes the SNP rs28416813-rev. The dashed line denotes the deletion made between +115 and +163 to generate ΔNF construct. The sequences of the other constructs used are shown in the right with the non-cosensus NF-κB site underlined and the SNP rs28416813-rev in italics. B) A representative plot (of at least two independent experiments, error bars show SD of triplicates) of luciferase activities of p1.4IL28B constructs with G or C alleles at rs28416813-rev and with different mutations introduced in to the non-consensus NF-κB site at +115 position to the plasmid with G allele background.

To further examine the role of the level of NF-κB binding at the non-consensus site on reporter gene expression, we used p1.4IL28BG-T to make three more constructs: 1) ΔNF- which has a 48-bp deletion of the DNA sequence including the non-consensus NF-κB-binding site up to the nucleotide for start codon (Fig. 6A), 2) Scr, which has a scrambled consensus NF-κB-binding sequence at the non-consensus site to eliminate NF-κB binding (Fig. 6A), and 3) Con, which replaces the non-consensus NF-κB site with a consensus NF-κB-binding site (Fig. 6A). Equal amounts of the respective plasmids were transfected in to HEK293T cells along with p50 and p65 expression plasmids and transfection controls and firefly luciferase activity was measured and normalized to *Renilla* luciferase levels (Fig. 6B). Surprisingly, ΔNF showed a substantial increase in luciferase activity (Fig. 6B). The Con construct also exhibited several-fold increase in luciferase activity whereas the Scr construct phenocopied the construct with the C allele at rs28416813-rev (Fig. 6B). This implies that the level of NF-κB binding at this distal promoter element of *IL28B* can significantly affect transcription of the *IL28B* gene. Furthermore, the result suggests that a C allele at rs28416813-rev decreases downstream gene expression most likely by decreasing NF-κB binding to the *IL28B* promoter.

### TNF-α and dsRNA can Stimulate the *IL28B* Promoter

**Figure 7:**
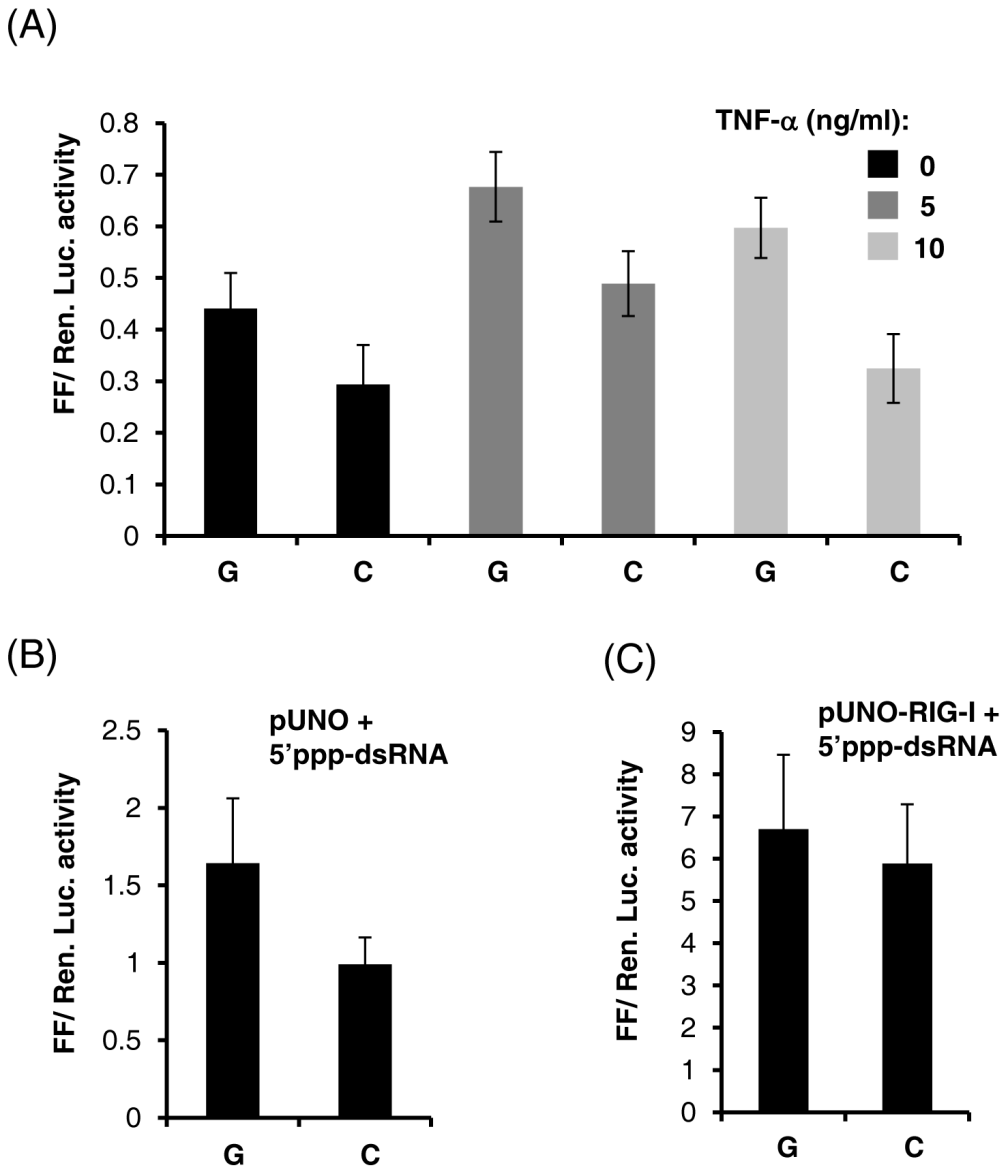
TNF-α and 5′ppp-dsRNA stimulate transcription from *IL28B* promoter. A) p1.4IL28B constructs (10 ng/well) with either G or C alleles at rs28416813-rev along with pRLTK were transfected in to 96-well plates and after 24 hr recombinant human TNF-α was added in to the media at indicated concentrations and plates were incubated for a further 8 hr before subjecting to luciferase assays. The experiment was done at least twice with similar results and the error bars show SD from triplicates. B) and C) p1.4IL28B constructs (10 ng/well) with either G or C alleles at rs28416813-rev along with pRLTK and either pUNO (B) or pUNO-RIG-I (C) were transfected in to 96-well plates and after 24 hr a 19-mer 5′ppp-dsRNA was transfected at 250 nM concentration. Luciferase assays were done after 12 hr. The error bars show SD of 6 replicates from two experiments.

So far we used exogenously expressed NF-κB to investigate how the two alleles at rs28416813-rev would influence transcription of the reporter gene. We next used an indirect strategy which will lead to stimulation of endogenous NF-κB by utilizing TNF-α (a well known NF-κB activator [27]) and synthetic dsRNA (19 mer) that is triphosphorylated at the 5′ end (a known ligand for RIG-I [28]). Both TNF-α and 5′ppp-dsRNA can stimulate the activation and translocation of NF-κB in to the nucleus by using different signaling pathways. TNF-α binds to its cognate receptor (TNFR1 and 2) and utilizes the mitogen activated kinase (MAPK) pathway to stimulate NF-κB activation and translocation. Moreover TNF-α has been found to be associated with chronic HCV infection [29]. TNF-α was added to the culture medium at two different concentrations 24 hr after the cells were transfected with p1.4IL28B constructs with the two alleles at rs28416813-rev and the pRLTK plasmid, and luciferase activity assessed after 8 hr (Fig. 7A). There was a stimulation of the promoter activity by TNF-α and the effect of the C allele in decreasing the transcription was evident (Fig. 7A).

Triphophorylated dsRNA is unique to RNA viral replication-intermediates and stimulates NF-κB by binding to RIG-I which then utilizes the mitochondrial membrane associated MAVS (mitochondrial antiviral signaling) protein for signal transduction [26]. Synthetic 5′ppp-dsRNA was transfected to cells 24 hr after they were transfected with p1.4IL28B constructs, pRLTK and either pUNO vector alone or pUNO-RIG-I, and luciferase activity was measured after 12 hr (Fig. 7B and 7C). RIG-I was able to increase the transcription of the *IL28B* promoter in presence of dsRNA and the difference in luciferase activity with the G and C alleles at rs28416813-rev was evident, albeit at a lower level when RIG-I was exogenously provided (Fig. 7B and 7C). These results show that the alternate alleles at rs28416813-rev can influence downstream gene transcription in presence of physiological inducers of NF-κB like TNF-α and dsRNA.

### HCV RNA-dependent RNA Polymerase (RdRp) can Stimulate *IL28B* Promoter

**Figure 8:**
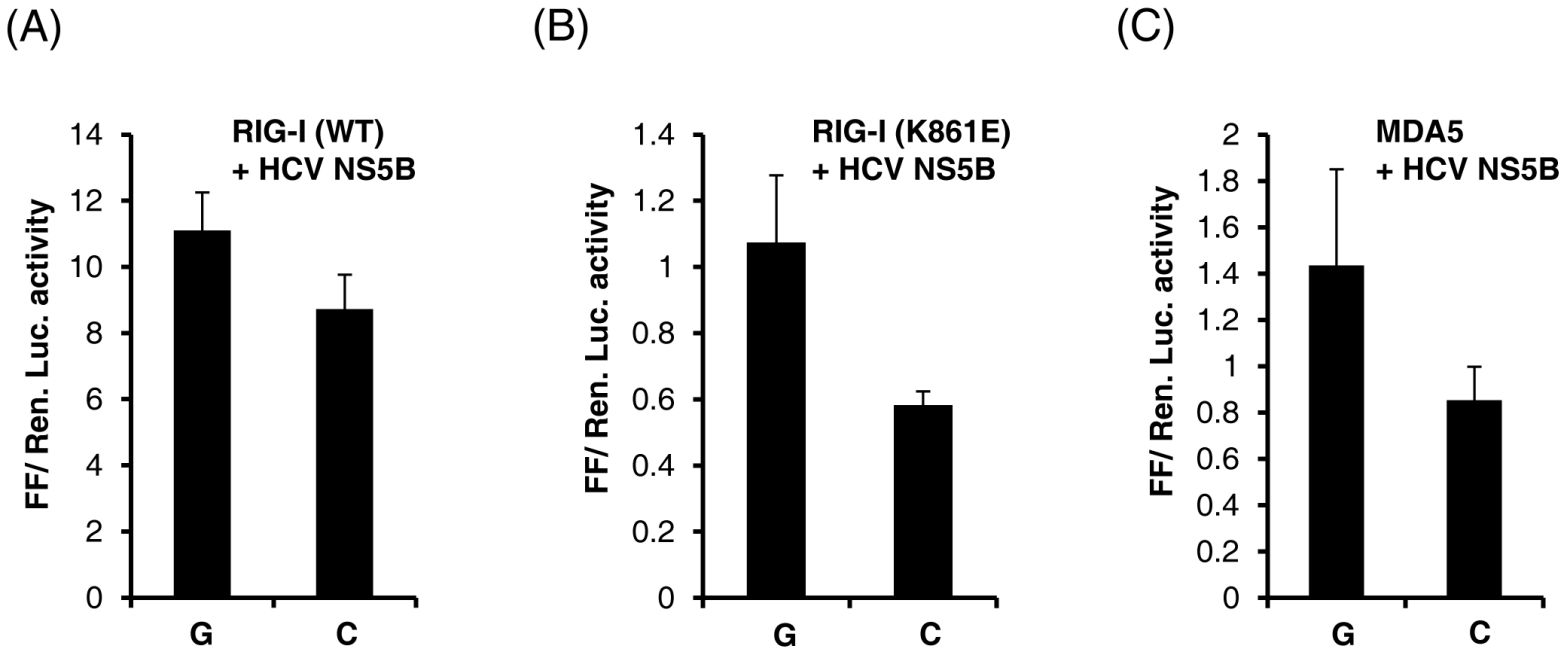
HCV RdRp can stimulate transcription from *IL28B* promoter. The gene encoding genotype 2a HCV RdRp (NS5B) was co-transfected at 50 ng/well along with the respective p1.4IL28B constructs at 10 ng/well and either RIG-I(WT) (A) RIG-I(K861E) (B) or MDA5 (C) at 0.5 ng/well [30], [31] along with pRLTK and luciferase assays were carried out after 36 hr. The experiment was repeated independently at least two times with similar results and the error bars show SD of triplicates from one experiment.

We next sought to determine the effect of the alternate alleles at rs28416813-rev on transcription in the context of HCV replication. We adapted a recently developed model system called the 5BR assay, developed by the Kao Lab [30] for this purpose. In this system HCV RdRp expressed within the cells (with no other HCV proteins) is capable of replicating viral and non-viral RNA within the cytoplasm and provide ligands for RIG-I to stimulate interferon-β promoter activity [30]. The ligands generated and the signaling induced by a viral RdRp through RIG-I is not unique to HCV but also has been shown for the norovirus RdRp [31]. Furthermore, the HCV RdRp activity within cells (with no other HCV proteins present) has been recently shown to be highly significant in generating innate immune responses in liver cells, in causing liver damage and in mediating pro-inflamatory responses that lead to tumorigenesis in both *in vitro* and *in vivo* mice models [32], [33]. We asked whether the ligands generated by HCV RdRp can stimulate *IL28B* promoter activity. As shown in Fig. 8 HCV RdRp (encoded by the viral gene NS5B) was able to stimulate the *IL28B* promoter about 10-fold in presence of the wildtype RIG-I (Fig. 8A). A mutant RIG-I (K861E) defective for RNA binding [30], [31] served as a control (Fig. 8B). MDA5 (melanoma differentiation-associated protein 5, a RIG-I like receptor that signals by binding to long dsRNA, [34]) could not replace RIG-I to stimulate *IL28B* promoter activity (Fig. 8C). While these results show that an active RdRp of HCV is enough to trigger *IL28B* promoter activity, more importantly they also confirm that the C allele at rs28416813-rev retains its inhibitory effect on gene transcription (Fig. 8).

### rs28416813 is in LD with the ‘Cosmopolitan’ SNP rs12979860

**Figure 9:**
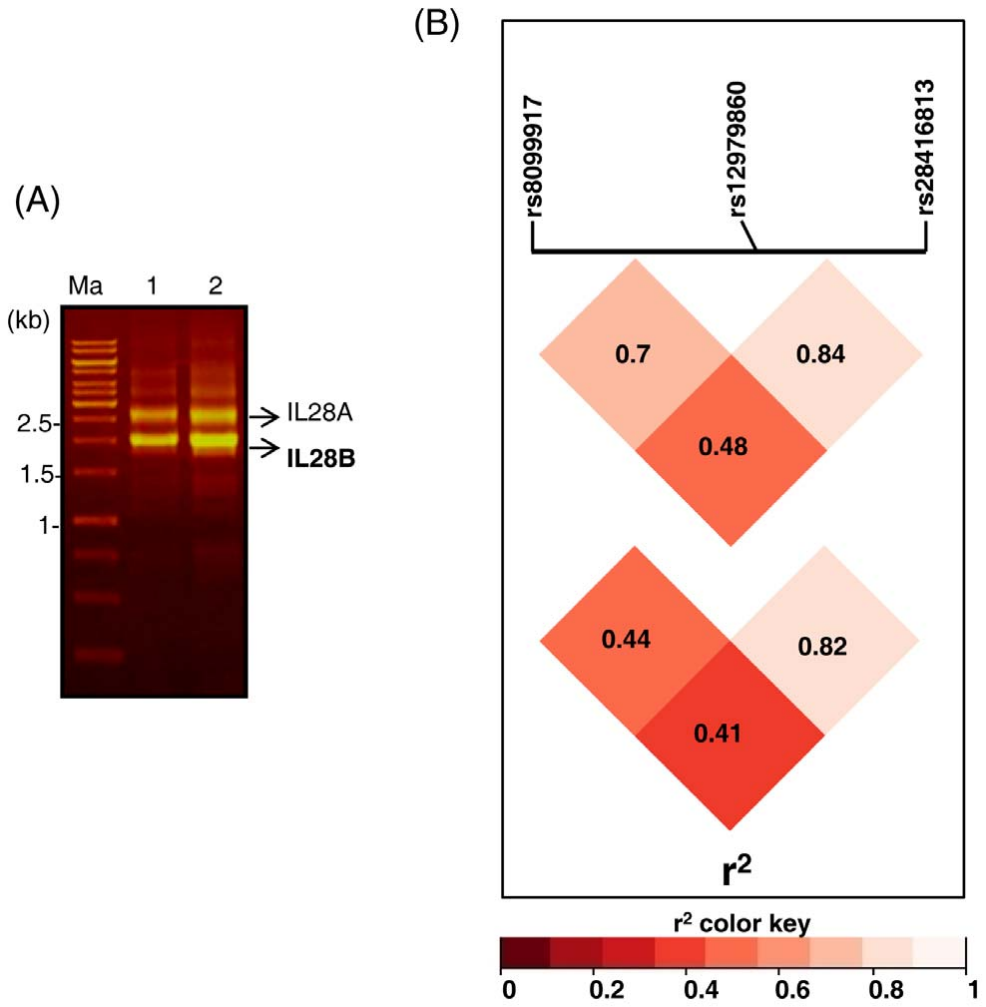
rs28416813 is in LD with rs12979860. A) EtBr-stained gel of the PCR products that were amplified from the *IL28A* and *IL28B* promoters that included the SNP rs28416813 by the same set of primers because of high homology between the two genes at their 5′ ends [35]. The ∼2 kb fragment specific to *IL28B* was excised from gels purified and subject to DNA sequencing to genotype the SNP rs28416813. 1 and 2 are samples from two different patients. Ma-DNA Mol. Wt. marker. B) Pairwise LD plots for the three SNPs genotyped from a cohort of patients with chronic HCV infections in India (n = 20) (top) and LD plots for the SNPs from genotype data of a CEU population available from the 1000 genomes project database (bottom). The plots were generated using LDHeatmap [20].

We genotyped a small group of patients with chronic HCV infections (n = 20) at the three SNP positions: rs12979860, rs8099917 and rs28416813. The first two SNPs were genotyped by DNA sequencing and RFLP methods using specific primer sets. However, for rs28416813 high level of homology between *IL28A* and *IL28B* genes towards their 5′ ends precludes independent amplification and genotyping [35]. In fact, the DNA sequence flanking the two SNP sites under study is identical between the duplicated genes except that the polymorphisms are seen only in *IL28B* whereas no variation is reported for *IL28A* (dbSNP, NCBI). Therefore, we designed primers such that the sizes of the two amplicons were different and then amplified both promoter regions using the same primer set (materials and methods). Following electrophoresis on EtBr-stained agarose gels (Fig. 9A) the *IL28B* amplicon was excised, purified and sequenced.

Extent of LD among the three SNP sites was estimated and found that the functional SNP rs28416813 is in high LD with the ‘cosmopolitan’ SNP rs12979860 (r^2^ = 0.84, D′ = 0.95, Fig. 9B, top) while it was not in LD with rs8099917 (r^2^ = 0.48, D′ = 0.85). We also estimated the LD between the three SNP positions from the CEU (Utah residents of northern and western European ancestry) population data available from the 1000 genomes project database [17]. Even in this population, the LD between the functional SNP rs28416813 and the ‘cosmopolitan’ SNP rs12979860 was high (r^2^ = 0.82, D′ = 1.0, Fig. 9B, bottom). These results suggest that the two SNPs co-segregate in different ethnic groups [11].

## Discussion

Although the GWAS in the treatment response to HCV infections and for spontaneous clearance of HCV has focused excitement in the field to the role of *IL28B* in disease outcome, it is not clear how the identified genetic variants affect *IL28B* expression [15]. Since 2009, many groups in different countries have replicated the associations of the SNPs at the *IL28B* locus with treatment response in chronic HCV infections [36]. The studies have been primarily focused on genotype 1 HCV infections as they are more prevalent in the western world and more difficult to treat. Since the lone SNP in the coding region of *IL28B* (rs8103142) did not apparently alter the function of the IFN-λ protein [13], [14], SNPs in the non-coding region may be playing a role in *IL28B* expression. The *IL28B* gene product (IFN-λ3 protein) is known to induce the expression of several hundreds of genes in a manner analogous to IFN-α [37]–[40]; hence even a modest change in *IL28B* levels could have amplified effects in the response to HCV infection [37]–[40]. No consensus currently exists about the expression levels of IFN-λ in PBMCs of responders versus non-responders to IFN-RBV treatment in chronic HCV infections although consistent findings on *IL28B* genotype-dependent differences in intrahepatic expression levels of interferon stimulated genes (ISGs) are being reported [15]. Some studies show that the protective or favorable *IL28B* alleles are associated with lower IFN-λ mRNA levels in liver biopsies from responders versus non-responders [41] while others have not observed this relationship [13], [42].

However, several reports are emerging in addition to previous ones [4], [5] on *IL28B* genotype-dependent expression/protein levels of lambda interferons [43], [44], [45]. Langhans *et al*., have recently shown that the favorable *IL28B* alleles are associated with significantly increased serum levels of *IL28A/B* and *IL29* gene products [43]. IFN-λ3 levels were significantly increased in patients who had spontaneously cleared HCV infections and carrying the favorable *IL28B* alleles compared to those with unfavorable alleles in a study from China [44]. The levels of IFN-λ3 after ex-vivo stimulation of PBMCs depend on the *IL28B* genotype and also determine treatment response to chronic HCV infections [45]. These reports do not show robust differences in the *IL28B* gene product levels between the different *IL28B* genotypes although being statistically significant [4], [5], [43], [44], [45], suggesting that subtle changes in *IL28B* gene expression is sufficient for an effect on the phenotype. This is likely because interferons are known to depend on feed-forward amplification of signals for their functions and small changes in gene expression will lead to large downstream effects [46]. Furthermore, the effect size of critical genes involved in complex diseases including chronic HCV infections is pretty low as evidenced by low to moderate odds ratios in association tests [3], [4], [5], [6], [47], implying that the effect of subtle changes in expression levels of these genes should be appreciated in the disease outcome.

While initially counterintuitive, it is likely that a lower level of hepatic ISG expression may favor a positive response to IFN-RBV treatment in chronic HCV infections [36]. This argument is especially relevant in the wake of the recent discovery of IFN-λ4 and its role in treatment response in chronic HCV infections [48]. In the report by Prokunina-Ollson *et al*., it was concluded that a novel IFN-λ was expressed only in non-responders which may be responsible for high levels of ISG expression seen in these patients [48]. Interestingly this novel interferon was not robustly secreted out in to the cell-culture medium and neither did a recombinant protein added to the medium stimulate transcription from an ISRE-luciferase (interferon stimulated response element) construct [48]. Inspite of this interesting discovery the role(s) of the other IFN-λs (1, 2 and 3) remain unclear in both spontaneous clearance and treatment response in chronic HCV infections. In this context our results show for the first time that an *IL28B* SNP influences downstream gene expression. We have shown that the G allele at rs28416813 (or the C allele in rs28416813-rev) reduces downstream reporter gene expression. The mechanism of the effect is likely through reduced NF-κB binding at the distal promoter element. A constant stimulation of innate immunity pattern recognition receptors particularly RIG-I and TLR-3 can lead to robust induction of *IL28* responses in hepatocytes and dendritic cells [49], [50]. However, the virus has devised mechanisms to blunt these responses [24], [51].

Our results with the ΔNF construct (Fig. 6B) suggest that the distal promoter element involving the non-consensus NF-κB site could act as a silencer. Furthermore, NF-κB binding at the non-consensus site slightly negates this silencing effect. The nearby SNP rs28416813 may play a role in allowing different levels of NF-κB binding at the non-consensus site leading to slightly different levels of inhibition of the silencer element. This will lead to different levels of *IL28B* expression in patients. Our results also suggest that the non-consensus NF-κB site identified by Osterlund *et. al.,* [21] is not the only NF-κB element in the *IL28B* promoter, but has other NF-κB-binding sites as identified by another recent study [24]. Our results clearly suggest that the site identified by the former group functions as a weak enhancer in presence of NF-κB. More experiments are needed to reveal the components and structure of the IFN-λ3 enhanceosome. A failure to see robust transcription of the *IL28B* promoter construct in Huh7.5 cells suggests that the IFN-λ3 enhanceosome has components other than NF-κB (Fig. 5B) that needs to be revealed to understand *IL28B* transcription better.

A recent study by the Mizokami lab examined the functional significance of several SNPs at the *IL28B* locus, including rs28416813 [14]. However, in that study, SNP rs28416813 was analyzed for effects on pre-mRNA splicing and not for its effect on transcription, and the conclusion was that splicing was not affected [14]. Since this SNP occurs close to a distal promoter element as shown in our study its effect on transcription is more likely to be important in the context of *IL28B* gene expression.

While the results presented in Fig. 7 demonstrate that the alleles at rs28416813 can affect the transcription of *IL28B* in conditions prevailing during HCV infection (TNF-α and 5′ppp-dsRNA both by-products of HCV infection), our results shown in Fig. 8 reveal the significance of our finding during active HCV replication. HCV RdRp has just been shown to be the key viral protein responsible for a pro-inflammatory response in HCV infections that may lead to tumorigenesis [33]. The polymerase activity of NS5B was required for activating NF-κB by both the canonical and alternate signaling pathways for this pro-inflamatory response [33]. In another recent study it was shown that transient expression of HCV NS5B alone in transgenic mice liver is enough to produce small RNA species to activate innate immune signaling involving NF-κB and IRF3 and subsequently cause liver damage [32]. These studies underlie the significance of the RdRp activity of NS5B in producing RNA ligands to activate innate immunity pathways within HCV infected cells. Our results (Fig. 8) clearly show that the presence of an active HCV RdRp in the cytoplasm generates signals to stimulate *IL28B* expression and that transcription at the *IL28B* promoter is influenced by the alleles present at rs28416813.

In summary we show that an SNP located in the distal promoter region of *IL28B*, influences downstream reporter gene expression due to its proximity to an NF-κB-binding site. The G (or C allele in rs28416813-rev) allele which is associated with an unfavorable treatment response to SOC treatment in chronic HCV infections [3], [5], [11] lowers the reporter gene expression compared to the favorable C allele (or G allele in rs28416813-rev). SNPs like rs12979860 and rs8099917 identified by GWA studies may be in high LD with rs28416813 and may exert their effect on the phenotype through rs28416813 (Fig. 9B), or they may have additional yet unidentified roles in *IL28B* expression and/or may be in LD with other SNPs. The results presented in this report strongly imply that changes in expression of *IL28B* at the level of transcription may affect spontaneous clearance of HCV and the treatment response to SOC therapy in chronic HCV infections. Our study shows how one of the SNPs at the *IL28B* locus may contribute to this process.
